# Structural birth defects and exposure to oil and gas wells during pregnancy

**DOI:** 10.1038/s41390-025-04719-1

**Published:** 2026-01-15

**Authors:** Juliana Stone, Jonathan A. Mayo, Gary M. Shaw, David J. X. Gonzalez

**Affiliations:** 1 Department of Pediatrics, School of Medicine, Stanford University, Stanford, CA USA; 2 Division of Environmental Health Sciences, School of Public Health, University of California, Berkeley, Berkeley, CA USA

## Abstract

**Background:**

Exposure to unconventional hydraulically fractured oil and gas wells during pregnancy is associated with a higher risk of structural birth defects. These associations have not been examined in California, where most operators use conventional methods that do not use enhanced techniques.

**Objectives:**

Determine whether residing near oil and gas wells during early pregnancy was associated with birth defect risks among San Joaquin Valley, California, residents.

**Methods:**

We conducted a case-control study with data from the California Center of the National Birth Defects Prevention Study for births delivered from 1997–2011. We considered 16 structural birth defect phenotypes. We assessed exposure to active wells within 3 km of the maternal residence or inactive wells within 1 km. We fit adjusted logistic regression models for each exposure and birth defect phenotype.

**Results:**

Exposure to active wells was associated with elevated odds of some CHDs and significantly lower odds of atrial septal defect secundum and gastroschisis. Exposure to inactive wells was associated with significantly elevated odds of cleft palate.

**Conclusions:**

Among San Joaquin Valley residents, living near active and inactive wells was associated with risks of birth defects. The estimates were imprecise and, given the number of statistical tests we conducted, may be spurious.

**Impact:**

- Researchers have not yet characterized structural birth defect-related potential risks associated with living near conventional wells in California, and there is little understanding of the hazards associated with inactive wells.
- We found that living near active wells in the San Joaquin Valley, California, was associated with risk of some structural birth defects, but findings were imprecise.
- Living near inactive wells was associated with an elevated risk of cleft lip and cleft palate.
- Further research could help determine whether findings were valid and explicate plausible etiological pathways.

## Introduction

In the United States (U.S.), approximately 3% of children have a major birth defect, a broad category that includes hundreds of phenotypes with multifactorial etiologies.^1^ Structural birth defects elevate risks of infant morbidity and mortality, and contribute to approximately 20% of infant deaths.^1^ Exposures to environmental contaminants during the perinatal period, including air and water pollution, contribute to genetic or teratogenic effects that lead to birth defects.^2^ Fetal susceptibility to teratogens may be heightened during the critical window that occurs just before conception and in the first 2 months of gestation, during organogenesis. There is robust evidence regarding the teratogenic potential of air and water pollutants produced during oil and gas production.^3–5^ Inactive wells also emit known teratogens, including benzene, toluene, ethylbenzene, and xylene (BTEX, collectively).^6^ However, despite these hazards, the potential associations of exposures to oil and gas development and risks of structural birth defects have not been well characterized.

The U.S. has been a leading global producer of oil and natural gas since 2014, and California is among the most productive states.^7–9^ Residential proximity to oil and gas development is associated with elevated risk of preterm birth, impaired fetal growth, and fetal loss.^10–16^ An estimated 17.6 million U.S. residents live within 1.6 km (1 mile) of an active oil and gas well, including 2.1 million Californians.^17^ Exposure to inactive wells, a broad category that includes idle and plugged wells, is even more widespread. Approximately 9 million Californians live within 1 km of inactive wells that have been plugged and abandoned.^18^ Prior work from Colorado, Texas, Oklahoma, and Ohio found that living near unconventional hydraulically fractured oil and gas wells is associated with elevated risks of congenital heart defects (CHDs), neural tube defects (NTDs), and oral clefts.^19–23^ In California, by contrast, the majority of oil and gas production uses conventional means not involving hydraulic fracturing.

In the current study, we examine risks of structural birth defects among people exposed to oil and gas development during pregnancy. We focused on the San Joaquin Valley, California, the most productive region for oil and gas in the state, which is predominated by conventional wells.

## Methods

### Study population

**Fig. 1:**
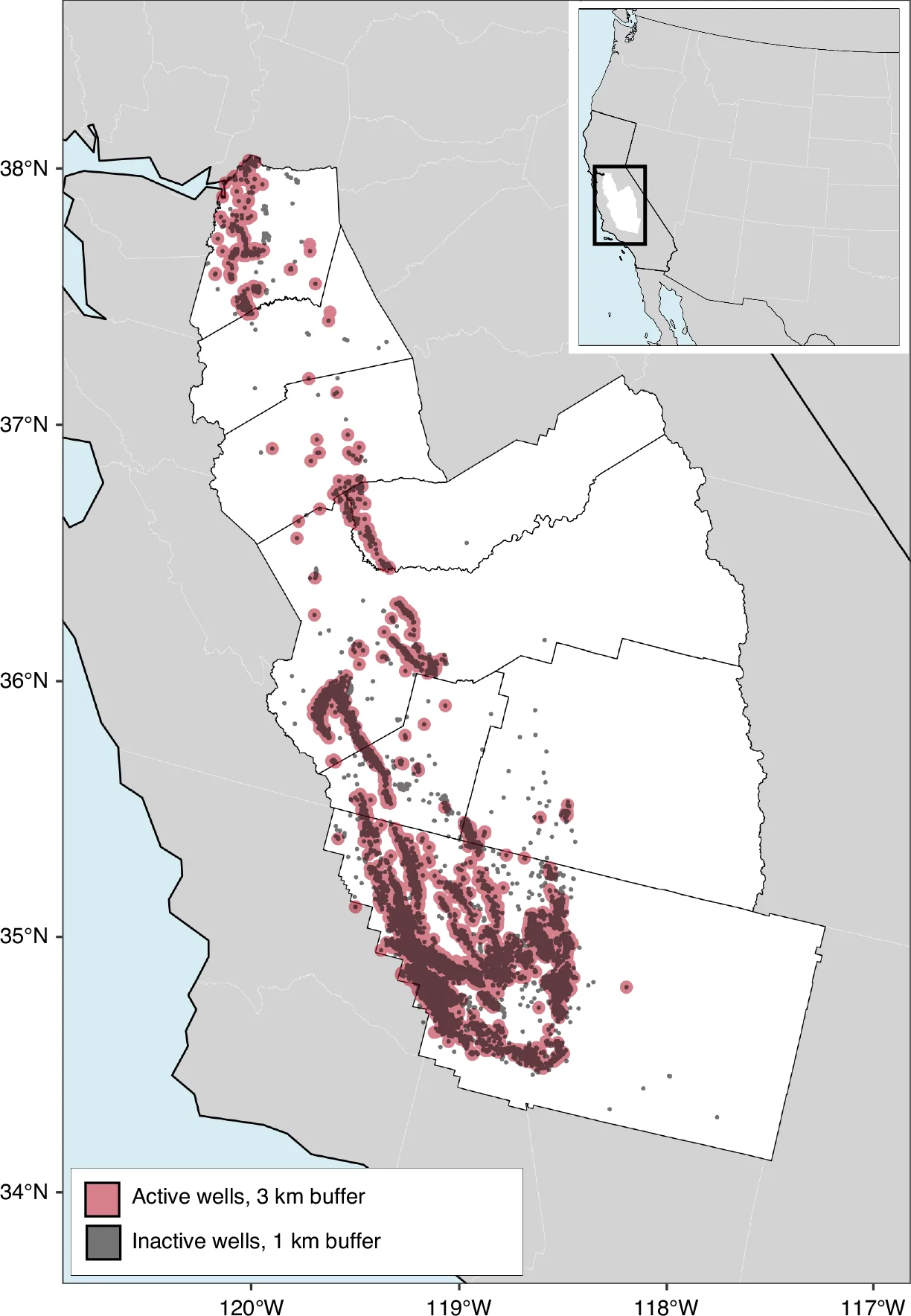
Map of study region (white background) showing a 3 km buffer around wells that were drilled or active during the study period (red). A 1 km buffer around wells that were inactive during the study period is shown in gray.

We conducted a case-control study among residents of the San Joaquin Valley, California. The study region comprised eight counties: San Joaquin, Stanislaus, Merced, Madera, Fresno, Kings, Tulare, and Kern (Fig. 1). The majority of oil and gas production in California occurs in Kern County. We obtained birth outcomes data from the California Center of the National Birth Defects Prevention Study (NBDPS), a collaborative partnership between Stanford University and the California Birth Defects Monitoring Program, a long-term population-based surveillance program. Study participants reported their residential history beginning 3 months before conception and continuing through delivery, for all residences occupied for at least 1 month, including dates when residence began and ended. Residences were geocoded to facilitate the exposure assessment described below. To identify cases with birth defects, Center staff visited all hospitals with obstetric or pediatric services, cytogenetic laboratories, and clinical genetics prenatal and postnatal outpatient services within the study region. Staff conducted interviews to gather additional information related to maternal health, pregnancy history, sociodemographic characteristics, and folic acid intake.^24^ Interviews were conducted primarily by telephone in English or Spanish at 6–24 months after delivery.

### Outcome variables

We considered 16 birth defect phenotypes. These included the NTDs anencephaly and spina bifida, as well as cleft palate and cleft lip. We also examined the conotruncal defects, Tetralogy of Fallot and transposition of the great arteries, and the CHDs aortic stenosis, secundum atrial septal defect (ASD), coarctation of the aorta, hypoplastic left heart syndrome, pulmonary valve stenosis, and perimembranous ventricular septal defect (VSD).

### Exposure assessment

We obtained data on oil and gas wells from the California Geologic Energy Management Division (CalGEM). The dataset included 239,383 wells of all types and at all stages of production, with latitude and longitude for each. We identified 47,446 wells that were active in the San Joaquin Valley from 1997 to 2011 and an additional 83,741 inactive wells. For the primary set of analyses, we defined exposure as residing within 3 km of an active well during the critical window, defined as 1 month pre-conception to 2 months post-conception. The 3 km threshold aligns with earlier studies investigating associations between exposure to oil and gas development and adverse birth outcomes.^25^ Prior environmental studies reported higher concentrations of criteria air pollutants within 3 km downwind of new and active wells.^4,5^ We defined unexposed as individuals who neither resided within 3 km of an active nor inactive well during their pregnancy.

In a separate set of analyses, we also assessed exposure only to inactive wells, a broad category that includes wells that are idle, plugged and abandoned, or abandoned and unplugged. More California residents are exposed to inactive than active wells, though in the San Joaquin Valley, exposure to active wells is correlated with exposure to inactive wells.^18^ For this set of analyses, we defined exposure as residing within 1 km of one or more inactive wells only, excluding those who also lived near active wells. Prior work has found elevated concentrations of teratogenic compounds within 1 km of inactive wells.^6^ As a sensitivity analysis, we also examined individuals living between 1 and 3 km from only inactive wells (data not shown).

### Inclusion criteria

For the current study, the study sample comprised deliveries between October 1, 1997, and December 31, 2011. The cases included infants and fetuses (stillbirths and elective terminations) with structural birth defects; we excluded those recognized or strongly suspected to have single gene mutations, chromosomal abnormalities, or with identifiable syndromes, given their presumed distinct underlying etiology. Because diabetes is associated with the risk of structural birth defects, we excluded births/fetuses delivered to individuals diagnosed with diabetes prior to pregnancy.^26^ Controls included non-malformed live births randomly selected from hospitals to represent the population from which the cases arose. Our analytic dataset was comprised of cases and controls that were either exposed to active wells within 3 km of the residence, exposed to inactive wells within 1 km of the residence, or births to individuals with no active and inactive wells within 3 km of the residence. We excluded records with missing observations for maternal age, educational attainment, or intake of folic acid-containing vitamins, which comprised 3.4% of births that otherwise met the inclusion criteria.

### Statistical analyses

To examine associations between our exposure to oil and gas wells and the 16 selected birth defects, we fit logistic regression models. In our primary analyses, we fit models adjusted for maternal age, educational attainment, and intake of folic acid-containing vitamin supplements from the month before conception to the 2 months following conception. The control group represents all births without major structural defects in the analytic dataset, and these served as the referent for each case group. We used an alpha value of 0.05 to assess statistical significance, as suggested by Rothman when making multiple comparisons.^27^ We used SAS version 9.4 (SAS Institute, Cary, NC) and R version 4.3.1 to prepare the data, assess exposure, and conduct statistical analyses.^28^

## Results

The analytic dataset comprised 2737 cases and 894 controls. Among cases and controls, the most common maternal age range at delivery was 20–24 years (Table 1). With respect to ethnic identity, 58% of maternal study participants were Hispanic. Among all study participants, 31% were foreign-born Hispanic and 27% were U.S.-born Hispanic, 2.5% were non-Hispanic Black, 29.4% were non-Hispanic white, 9.9% had a different racial/ethnic identity, and 0.1% had missing racial/ethnic identifiers. Within the study population, 30.9% had not completed high school, 29.5% had completed high school, and 39.6% had post-secondary education. Regarding parity, 37.6% of births were primiparous, 30.2% were to individuals with one prior birth, and 32.2% were to individuals with two or more prior births. Among study participants, 67.2% took folic acid supplements during the month prior to conception through the first 2 months of pregnancy; 43.7% of infants were female, and 55.9% were male.

**Table 1:** Characteristics of the pregnant individuals in the study population, San Joaquin Valley, California.

Characteristic	All	Cases	Controls	Active wells within 3 km	Inactive wells within 1 km	Unexposed
*n*	3631	2737	894	414	179	3038
Age at delivery, *n* (%)						
≤19	545 (15.0)	406 (14.8)	139 (15.5)	59 (14.3)	25 (14.0)	461 (15.2)
20–24	1039 (28.6)	781 (28.5)	258 (28.9)	138 (33.3)	63 (35.2)	838 (27.6)
25–29	972 (26.8)	734 (26.8)	238 (26.6)	104 (25.1)	43 (24.0)	825 (27.2)
30–34	700 (19.3)	530 (19.4)	170 (19.0)	80 (19.3)	25 (14.0)	595 (19.6)
≥35	375 (10.3)	286 (10.4)	89 (10.0)	33 (8.0)	23 (12.8)	319 (10.5)
Race/Ethnicity, *n* (%)						
Hispanic, Foreign Born	1127 (31.0)	864 (31.6)	263 (29.4)	86 (20.8)	80 (44.7)	961 (31.6)
Hispanic, U.S. Born	981 (27.0)	744 (27.2)	237 (26.5)	115 (27.8)	47 (26.3)	819 (27.0)
Non-Hispanic Black	92 (2.5)	66 (2.4)	26 (2.9)	20 (4.8)	3 (1.7)	69 (2.3)
Non-Hispanic White	1068 (29.4)	785 (28.7)	283 (31.7)	163 (39.4)	26 (14.5)	879 (28.9)
Other	359 (9.9)	275 (10.0)	84 (9.4)	29 (7.0)	23 (12.8)	307 (10.1)
Missing	4 (0.1)	3 (0.1)	1 (0.1)	1 (0.2)	0 (0)	3 (0.1)
Education, *n* (%)						
<High School	1122 (30.9)	863 (31.5)	259 (29.0)	101 (24.4)	63 (35.2)	958 (31.5)
High School	1070 (29.5)	817 (29.9)	253 (28.3)	128 (30.9)	61 (34.1)	881 (29.0)
>High School	1439 (39.6)	1057 (38.6)	382 (42.7)	185 (44.7)	55 (30.7)	1199 (39.5)
Parity, *n* (%)						
Primiparous	1365 (37.6)	1034 (37.8)	331 (37.0)	143 (34.5)	62 (34.6)	1160 (38.2)
1 prior birth	1097 (30.2)	819 (29.9)	278 (31.1)	142 (34.3)	47 (26.3)	908 (29.9)
2 or more prior births	1169 (32.2)	884 (32.3)	285 (31.9)	129 (31.2)	70 (39.1)	970 (31.9)
Folic acid intake, *n* (%)						
No	1190 (32.8)	902 (33.0)	288 (32.2)	123 (29.7)	59 (33.0)	1008 (33.2)
Yes	2441 (67.2)	1835 (67.0)	606 (67.8)	291 (70.3)	120 (67.0)	2030 (66.8)
Infant sex, *n* (%)						
Female	1588 (43.7)	1151 (42.1)	437 (48.9)	175 (42.3)	78 (43.6)	1335 (43.9)
Male	2028 (55.9)	1571 (57.4)	457 (51.1)	236 (57.0)	100 (55.9)	1692 (55.7)
Missing	15 (0.4)	15 (0.5)	0 (0)	3 (0.7)	1 (0.6)	11 (0.4)

Percentages are for each column.

Among all study participants, 414 (11.4%) lived within 3 km of active wells, and an additional 179 (4.9%) lived within 1 km of inactive wells. There were 3038 (83.7%) unexposed participants who did not live near any type of well and served as the referent group. Compared to unexposed individuals, those living within 3 km of active wells were more likely to be younger (<25 years old) and white (non-Hispanic). Participants exposed to inactive wells within 1 km were also more likely to be younger than unexposed participants.

**Fig. 2:**
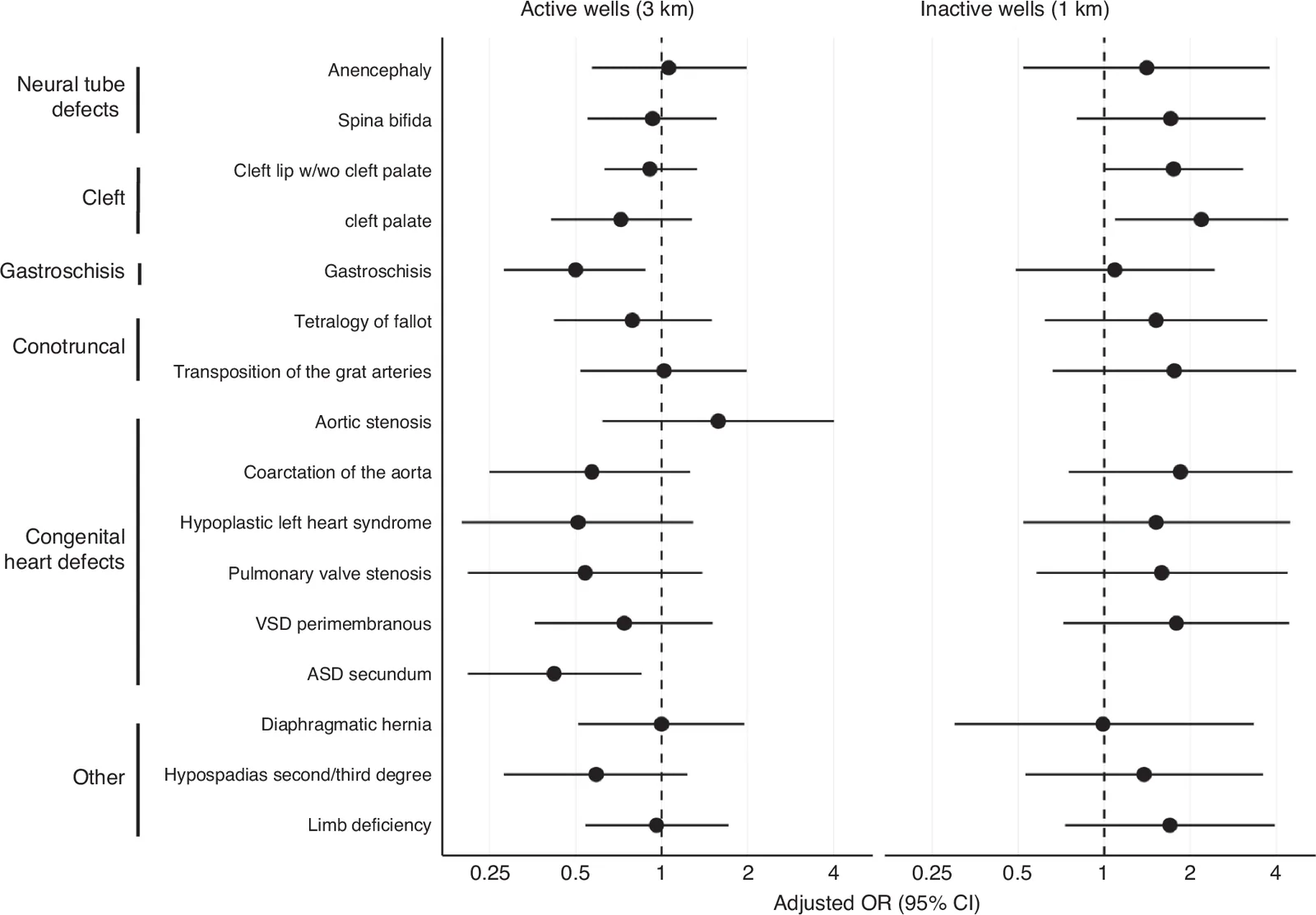
Adjusted odds ratios (OR) and 95% confidence intervals (CI) for the association between each birth defect phenotype and (left) exposure to active wells within 3 km of the residence and (right) inactive wells only within 1 km of the residence. ASD atrial septal defect, VSD ventricular septal defect. Note that the *x*-axis is on a log scale.

In adjusted models, we observed that exposure to active wells within 3 km of the residence was associated with reduced odds of gastroschisis and secundum ASD (Fig. 2). We did not observe any statistically significant positive associations for any of the structural birth defects we considered (Table S1). Exposure to active wells was associated with elevated odds of aortic stenosis, though this estimate is not statistically significant. There were numerous associations with lower odds, including for cleft palate, hypospadias second/third degree, and the CHDs, including coarctation of the aorta, hypoplastic left heart syndrome, pulmonary valve stenosis, and perimembranous VSD. For each outcome and exposure, the crude estimates were similar to the adjusted estimates.

Living within 1 km of only inactive wells was associated with significantly elevated odds of cleft palate and cleft lip. There were no other statistically significant associations, and estimates were imprecise, but we observed elevated odds of spina bifida, limb deficiency, the conotruncal defects tetralogy of Fallot and transposition of the great arteries, and CHDs, including coarctation of the aorta, hypoplastic left heart syndrome, pulmonary valve stenosis, and perimembranous VSD.

In the sensitivity analysis examining exposures to those living within 1–3 km of only inactive wells, we observed higher odds of Ebstein anomaly and reduced odds of transposition of the great arteries, hypospadias, second/third degree, and pulmonary valve stenosis (Fig. S1).

## Discussion

We investigated whether people living near oil and gas wells in the San Joaquin Valley, California, had an elevated risk of structural birth defects, using a detailed dataset assembled by the California Center of the NBDPS. Exposure to active wells during pregnancy was associated with decreased odds of gastroschisis and secundum ASD. We also observed some indication of higher risks for several other CHD phenotypes with exposure to active wells, though none of these results were statistically significant. Our findings were mixed and imprecise but indicate potential associations that should be followed up on in further work.

Prior work has reported protective associations for a birth defect with exposure to oil and gas operations. Gaughan et al. found that living near unconventional oil and gas wells during pregnancy was associated with a significantly reduced risk of hypospadias.^19^ Given the suspected exposures that may be present near such operations—including BTEX, ozone, and fine particulate matter—to our knowledge, there is no evidence of any plausible salutogenic factors related to living near oil and gas wells and risk of gastroschisis and CHD.^4,5,29^ The protective associations we observed for gastroschisis and secundum ASD may be attributable to residual confounding. Another hypothesis that could explain these findings would be selective (for certain defects) culling of susceptible fetuses *in utero* before ascertainment, which would not be observable in the birth cohort data used in the current study. Future researchers could utilize longitudinal pregnancy cohorts to determine whether there is an association between exposures to oil and gas development during pregnancy and fetal loss.

Here, we built on prior work that primarily focused on regions with active hydraulically fractured wells. In California, most wells use conventional means (i.e., do not use hydraulic fracturing), and across the state, more people live near inactive wells than live near active wells. In 2019, an estimated 1.1 million California residents lived within 1 km of active wells, whereas an estimated 9.0 million lived within 1 km of inactive, plugged wells.^18^ Despite their widespread distribution near inhabited areas, inactive wells have received little attention from public health researchers. There has, however, been interest in abandoned wells from researchers focused on averting greenhouse gas emissions. For example, Kang et al. report that some abandoned wells in Pennsylvania emitted substantial volumes of methane.^30^ Methane is often co-emitted with non-methane volatile organic compounds, and a 2023 study—also in Pennsylvania—found elevated concentrations of benzene and other non-methane VOCs near abandoned well sites.^6^ Exposure to benzene was previously found to be associated with a higher risk of cleft palate.^31^ We only observed this association for people near inactive wells, and not among people living further (1–3 km) from inactive wells or exposed to active wells. Further work could help determine whether this is a consistent association, identify plausible etiologic pathways, and examine potential drivers underlying this health disparity. Researchers should also examine whether living near inactive wells is associated with other adverse birth outcomes. Nearly one quarter of the 3.2 million abandoned wells in the U.S. are located within 100 m of structures, indicating widespread potential for human exposure.^6^

Prior studies from Canada and the U.S. reported that living near oil and gas development was associated with increased risks of CHDs, NTDs, gastroschisis, limb reduction defects, and oral clefts. The methods used by these researchers to assess exposures to oil and gas development varied, which makes it difficult to draw direct comparisons. Researchers in Colorado report that living within a 10-mile radius of natural gas development during pregnancy was associated with significantly increased odds of CHDs and increased prevalence of NTDs among births with the highest exposure, although they didn’t account for folic acid intake and co-occurring genetic anomalies.^23^ In Texas, Tang et al. found that relatively high exposure to unconventional natural gas wells was associated with higher risk of anencephaly, spina, gastroschisis among older mothers, aortic valve stenosis, hypoplastic left heart syndrome, and pulmonary valve atresia or stenosis.^21^ Another study found that cardiac and circulatory defects were strongly and consistently associated with exposure to hydraulically fractured wells.^32^ An Oklahoma study reported higher rates of CHD, NTD, and oral clefts with exposure to natural gas activity during the month of delivery and found increased, though imprecise, prevalence of NTDs among children with natural gas activity compared to unexposed children.^22^ In Alberta, Canada, Cairncross et al. expanded the exposure time period to 1 year prior to conception through delivery date and found major birth defects as an overall grouping were significantly higher in individuals who lived within 10 km of at least 1 hydraulically fractured well after adjusting for parental age at delivery, multiple births, fetal sex, obstetric complications, and area level socioeconomic status.^33^ In Ohio, where a 30-fold increase in natural gas production occurred in the past decade, researchers found elevated risk for NTDs, limb reduction defects, and spina bifida among infants born to individuals living within 10 km of unconventional oil and gas development, compared to unexposed pregnancies.^19^

The precise mechanisms by which teratogenic exposures contribute to structural birth defects are still emerging. Specific agents often produce predictable lesions, although variation between individuals exists based on underlying genetics. Some agents have a dose or threshold effect, below which no negative effects are seen. In the literature to date, various studies have looked at associations between individual chemicals—many of which are byproducts of oil and gas production—and the development of the most common birth defects: Benzene and CHDs^34–36^; NO_2_, SO2 and CHD^37^; organic solvents and CHD^38–40^; PAH and NTD^41^; CO, O_3_ and NTD^41^; PM and CHD.^31,42,43^ When it comes to NTDs, Tang et al. and others have posited that acute, frequent, and concentrated airborne exposures from high-intensity production may put fetuses at the highest risk of NTDs, as the strongest associations were found in populations living within the closest radius to oil and gas wells.^21^ Pollutants such as benzene and PAH have been found to cross the placenta and, in certain cases, can be found in higher levels in the fetus than the mother.^44^ Pollutants can contribute to oxidative stress, DNA damage, formation of placental DNA adducts, and a proinflammatory state, among others.^44^

Given the numerous statistical associations we report, under the null hypothesis, we would expect that there would be some spurious associations. Consequently, we cannot reject the null hypothesis that there is no association between exposures to oil and gas wells and risk of structural birth defects. Further work with large sample sizes could help understand whether the reported associations were indeed spurious. Other limitations of the current study include assessing exposure to wells only at the place of residence, and not at other settings where participants spend time. We used a binary indicator of exposure in our metrics, and consequently, we were unable to account for differences among individuals exposed to many wells or wells that are particularly hazardous due to, for example, higher rates of emissions of ambient air pollutants. As in prior studies investigating exposures to oil and gas development, we were unable to ascertain which exact chemical compound was at play, and we did not utilize biological samples to precisely know exposure levels in the mother and fetus. We were also unable to account for race/ethnicity in our analyses, as we had insufficient statistical power to conduct race/ethnicity-stratified analyses to test for effect modification. Future research should assess for such effect modification. Strengths include our use of a well-established birth defects program, allowing for more complete case ascertainment. The dataset also included full residential history throughout the critical window of fetal development, which allowed for more accurate exposure assessment than assessing only at the residence at the time of delivery, as typically reported on birth certificates.

## Conclusion

We investigated whether living near oil and gas wells during pregnancy was associated with risk of structural birth defects in fetuses and infants delivered in the San Joaquin Valley, California. Prior studies in other settings observed higher risk of some birth defects was associated with living near oil and gas wells.^45^ In the current study, we observed that exposure to active wells was associated with elevated odds of some CHDs and significantly lower odds of ASD secundum and gastroschisis, and that exposure to inactive wells was associated with significantly elevated odds of cleft palate. The estimates were notably imprecise and, given the number of statistical tests we conducted, may be spurious. Further research could help determine whether these findings were valid and explicate plausible etiological pathways.

## Supplementary information

Supplementary Information

## Data Availability

Data obtained from the California Center of the NBDPS include protected health information and are not available for public use. Data for oil and gas wells are publicly available from the California Geologic Energy Management Division at https://www.conservation.ca.gov/calgem/maps/Pages/GISMapping2.aspx.^46^

## References

[1] Rynn, L., Cragan, J. & Correa, A. Update on overall prevalence of major birth defects—Atlanta, Georgia, 1978–2005. Division of Birth Defects and Developmental Disabilities, National Center on Birth Defects and Developmental Disabilities, CDC. [cited 2025 Apr 17]. Available from: https://www.cdc.gov/mmwr/preview/mmwrhtml/mm5701a2.htm (2008).

[2] Baldacci, S. et al. Environmental and individual exposure and the risk of congenital anomalies: a review of recent epidemiological evidence. *Epidemiol. Prev.* **42**, 1–34 (2018). 10.19191/EP18.3-4.S1.P001.057 30066535

[3] Elliott, E. G., Ettinger, A. S., Leaderer, B. P., Bracken, M. B. & Deziel, N. C. A systematic evaluation of chemicals in hydraulic-fracturing fluids and wastewater for reproductive and developmental toxicity. *J. Expo. Sci. Environ. Epidemiol.* **27**, 90–9 (2017). 10.1038/jes.2015.81 26732376

[4] Gonzalez, D. J. X. et al. Upstream oil and gas production and ambient air pollution in California. *Sci. Total Environ.* **806**, 150298 (2022). 10.1016/j.scitotenv.2021.150298 34844318

[5] Garcia-Gonzales, D. A., Shonkoff, S. B. C., Hays, J. & Jerrett, M. Hazardous air pollutants associated with upstream oil and natural gas development: a critical synthesis of current peer-reviewed literature. *Annu. Rev. Public Health* **40**, 283–304 (2019). 10.1146/annurev-publhealth-040218-043715 30935307

[6] DiGiulio, D. C. et al. Chemical characterization of natural gas leaking from abandoned oil and gas wells in western Pennsylvania. *ACS Omega* **8**, 19443–54 (2023). 10.1021/acsomega.3c00676 PMC10249135 37305312

[7] U.S. Energy Information Administration. Petroleum and other liquids: california field production of crude oil. [cited 2025 Apr 16]. Available from: https://www.eia.gov/dnav/pet/hist/LeafHandler.ashx?n=PET&s=MCRFPCA2&f=M (2025).

[8] U.S. Energy Information Administration. Natural gas. [cited 2025 Apr 16]. Available from: https://www.eia.gov/international/data/world/natural-gas/dry-natural-gas-production (2025).

[9] U.S. Energy Information Administration. Which countries are the top producers and consumers of oil?.[cited Apr 16]. Available from: https://www.eia.gov/tools/faqs/faq.php?id=709&t=6 (2024).

[10] Gonzalez, D. J. X. et al. Oil and gas production and spontaneous preterm birth in the San Joaquin Valley, CA: a case-control study. *Environ. Epidemiol.* **4**, e099 (2020). 10.1097/EE9.0000000000000099 PMC7423522 32832838

[11] Caron-Beaudoin, É. et al. Density and proximity to hydraulic fracturing wells and birth outcomes in Northeastern British Columbia, Canada. *J. Expo. Sci. Environ. Epidemiol.* 10.1038/s41370-020-0245-z (2020). 32651474

[12] Casey, J. A. et al. Unconventional natural gas development and birth outcomes in Pennsylvania, USA. *Epidemiology* **27**, 163–172 (2016). 10.1097/EDE.0000000000000387 PMC4738074 26426945

[13] Cushing, L. J., Vavra-Musser, K., Chau, K., Franklin, M. & Johnston, J. E. Flaring from unconventional oil and gas development and birth outcomes in the Eagle Ford Shale in south Texas. *Environ. Health Perspect.* **128**, 77003 (2020). 10.1289/EHP6394 PMC7362742 32673511

[14] Stacy, S. L. et al. Perinatal outcomes and unconventional natural gas operations in Southwest Pennsylvania. *PLoS ONE* **10**, e0126425 (2015). 10.1371/journal.pone.0126425 PMC4454655 26039051

[15] Tran, K. V., Casey, J. A., Cushing, L. J. & Morello-Frosch, R. Residential proximity to oil and gas development and birth outcomes in California: a retrospective cohort study of 2006-2015 births. *Environ. Health Perspect.* **128**, 067001 (2020). 10.1289/EHP5842 PMC7268907 32490702

[16] Whitworth, K. W., Marshall, A. K. & Symanski, E. Maternal residential proximity to unconventional gas development and perinatal outcomes among a diverse urban population in Texas. *PLoS ONE* **12**, e0180966 (2017). 10.1371/journal.pone.0180966 PMC5522007 28732016

[17] Czolowski, E. D., Santoro, R. L., Srebotnjak, T. & Shonkoff, S. B. C. Toward consistent methodology to quantify populations in proximity to oil and gas development: a national spatial analysis and review. *Environ. Health Perspect.* **125**, 086004 (2017). 10.1289/EHP1535 PMC5783652 28858829

[18] González, D. J. X. et al. Temporal trends of racial and socioeconomic disparities in population exposures to upstream oil and gas development in California. *GeoHealth* **7**, e2022GH000690 (2023). 10.1029/2022GH000690 PMC10035325 36968155

[19] Gaughan, C. et al. Residential proximity to unconventional oil and gas development and birth defects in Ohio. *Environ. Res.* **229**, 115937 (2023). 10.1016/j.envres.2023.115937 PMC10198955 37076028

[20] McKenzie, L. M. et al. Birth outcomes and maternal residential proximity to natural gas development in rural Colorado. *Environ. Health Perspect.* **122**, 412–7 (2014). 10.1289/ehp.1306722 PMC3984231 24474681

[21] Tang, I. W., Langlois, P. H. & Vieira, V. M. Birth defects and unconventional natural gas developments in Texas, 1999–2011. *Environ. Res.* **194**, 110511 (2020). 10.1016/j.envres.2020.110511 33245885

[22] Janitz, A. E., Dao, H. D., Campbell, J. E., Stoner, J. A. & Peck, J. D. The association between natural gas well activity and specific congenital anomalies in Oklahoma, 1997–2009. *Environ. Int.* **122**, 381–8 (2019). 10.1016/j.envint.2018.12.011 PMC6328052 30551805

[23] McKenzie, L. M., Allshouse, W. & Daniels, S. Congenital heart defects and intensity of oil and gas well site activities in early pregnancy. *Environ. Int.* **132**, 104949 (2019). 10.1016/j.envint.2019.104949 31327466

[24] Willett, W. C. et al. Reproducibility and validity of a semiquantitative food frequency questionnaire. *Am. J. Epidemiol.* **122**, 51–65 (1985). 10.1093/oxfordjournals.aje.a114086 4014201

[25] Deziel, N. C. et al. Assessing exposure to unconventional oil and gas development: strengths, challenges, and implications for epidemiologic research. *Curr. Environ. Health Rep.* 10.1007/s40572-022-00358-4 (2022). PMC9363472 35522388

[26] Carmichael, S. L., Rasmussen, S. A. & Shaw, G. M. Prepregnancy obesity: a complex risk factor for selected birth defects: prepregnancy obesity. *Birth Defects Res. A Clin. Mol. Teratol.* **88**, 804–10 (2010). 10.1002/bdra.20679 20973050

[27] Rothman, K. No adjustments are needed for multiple comparisons. *Epidemiology* **1**, 43–46 (1990). 2081237

[28] R Core Team. R: a language and environment for statistical computing. R Foundation for Statistical Computing. https://www.R-project.org/ (2022).

[29] Garcia-Gonzales, D. A., Shamasunder, B. & Jerrett, M. Distance decay gradients in hazardous air pollution concentrations around oil and natural gas facilities in the city of Los Angeles: a pilot study. *Environ. Res.* **173**, 232–6 (2019). 10.1016/j.envres.2019.03.027 30928853

[30] Kang, M. et al. Direct measurements of methane emissions from abandoned oil and gas wells in Pennsylvania. *Proc. Natl. Acad. Sci. USA* **111**, 18173–7 (2014). 10.1073/pnas.1408315111 PMC4280601 25489074

[31] Tanner, J. P. et al. Associations between exposure to ambient benzene and PM(2.5) during pregnancy and the risk of selected birth defects in offspring. *Environ. Res.* **142**, 345–53 (2015). 10.1016/j.envres.2015.07.006 26196779

[32] Willis, M. D., Carozza, S. E. & Hystad, P. Congenital anomalies associated with oil and gas development and resource extraction: a population-based retrospective cohort study in Texas. *J. Expo. Sci. Environ. Epidemiol.* **33**, 84–93 (2023). 10.1038/s41370-022-00505-x PMC9852077 36460921

[33] Cairncross, Z. F. et al. Association between residential proximity to hydraulic fracturing sites and adverse birth outcomes. *JAMA Pediatr.* **176**, 585–92 (2022). 10.1001/jamapediatrics.2022.0306 PMC8981068 35377398

[34] Lupo, P. J. et al. Maternal exposure to ambient levels of benzene and neural tube defects among offspring: Texas, 1999-2004. *Environ. Health Perspect.* **119**, 397–402 (2011). 10.1289/ehp.1002212 PMC3060005 20923742

[35] Badham, H. J., Renaud, S. J., Wan, J. & Winn, L. M. Benzene-initiated oxidative stress: effects on embryonic signaling pathways. *Chem. Biol. Interact.* **184**, 218–21 (2010). 10.1016/j.cbi.2009.11.005 19913523

[36] Wennborg, H., Magnusson, L. L., Bonde, J. P. & Olsen, J. Congenital malformations related to maternal exposure to specific agents in biomedical research laboratories. *J. Occup. Environ. Med.* **47**, 11–9 (2005). 10.1097/01.jom.0000150237.67801.93 15643154

[37] Vrijheid, M. et al. Ambient air pollution and risk of congenital anomalies: a systematic review and meta-analysis. *Environ. Health Perspect.* **119**, 598–606 (2011). 10.1289/ehp.1002946 PMC3094408 21131253

[38] Brender, J., Suarez, L., Hendricks, K., Baetz, R. A. & Larsen, R. Parental occupation and neural tube defect-affected pregnancies among Mexican Americans. *J. Occup. Environ. Med.* **44**, 650–6 (2002). 10.1097/00043764-200207000-00011 12138876

[39] Desrosiers, T. A. et al. Maternal occupational exposure to organic solvents during early pregnancy and risks of neural tube defects and orofacial clefts. *Occup. Environ. Med.* **69**, 493–9 (2012). 10.1136/oemed-2011-100245 PMC3719396 22447643

[40] McMartin, K. I., Chu, M., Kopecky, E., Einarson, T. R. & Koren, G. Pregnancy outcome following maternal organic solvent exposure: a meta-analysis of epidemiologic studies. *Am. J. Ind. Med.* **34**, 288–92 (1998). 10.1002/(sici)1097-0274(199809)34:3<288::aid-ajim12>3.0.co;2-q 9698999

[41] Naufal, Z. et al. Biomarkers of exposure to combustion by-products in a human population in Shanxi, China. *J. Expo. Sci. Environ. Epidemiol.* **20**, 310–9 (2010). 10.1038/jes.2009.19 19277067

[42] Girguis, M. S. et al. Maternal exposure to traffic-related air pollution and birth defects in Massachusetts. *Environ. Res.* **146**, 1–9 (2016). 10.1016/j.envres.2015.12.010 PMC4761511 26705853

[43] Ren, Z. et al. Maternal exposure to ambient PM10 during pregnancy increases the risk of congenital heart defects: evidence from machine learning models. *Sci. Total Environ.* **630**, 1–10 (2018). 10.1016/j.scitotenv.2018.02.181 29471186

[44] Wilbur, S., Keith, S., Faroon, O. & Wohlers, D. Toxicological profile for benzene. U.S. Department of Health and Human Services: Agency for Toxic Substances and Disease Registry. Available from: http://www.crcnetbase.com/doi/10.1201/9781420061888_ch38 (2007). 24049861

[45] Deziel, N. C. et al. Applying the hierarchy of controls to oil and gas development. *Environ. Res. Lett.* **17**, 071003 (2022). 10.1088/1748-9326/ac7967 PMC9328576 35910067

[46] California Geologic Energy Management Division (CalGEM). WellSTAR. [cited 2024]. Available from: https://wellstar-public.conservation.ca.gov/General/Home/PublicLanding (2024).

