# Supplementary material for: Structural birth defects and exposure to oil and gas wells during pregnancy
Source: Pediatr Res. 2026 Jan 15;100(3):1122–8. doi: 10.1038/s41390-025-04719-1 (PMC13672117; doi:10.1038/s41390-025-04719-1)
Supplement: Supplementary file 1 [file 41390_2025_4719_MOESM1_ESM.pdf]

Supplementary Materials

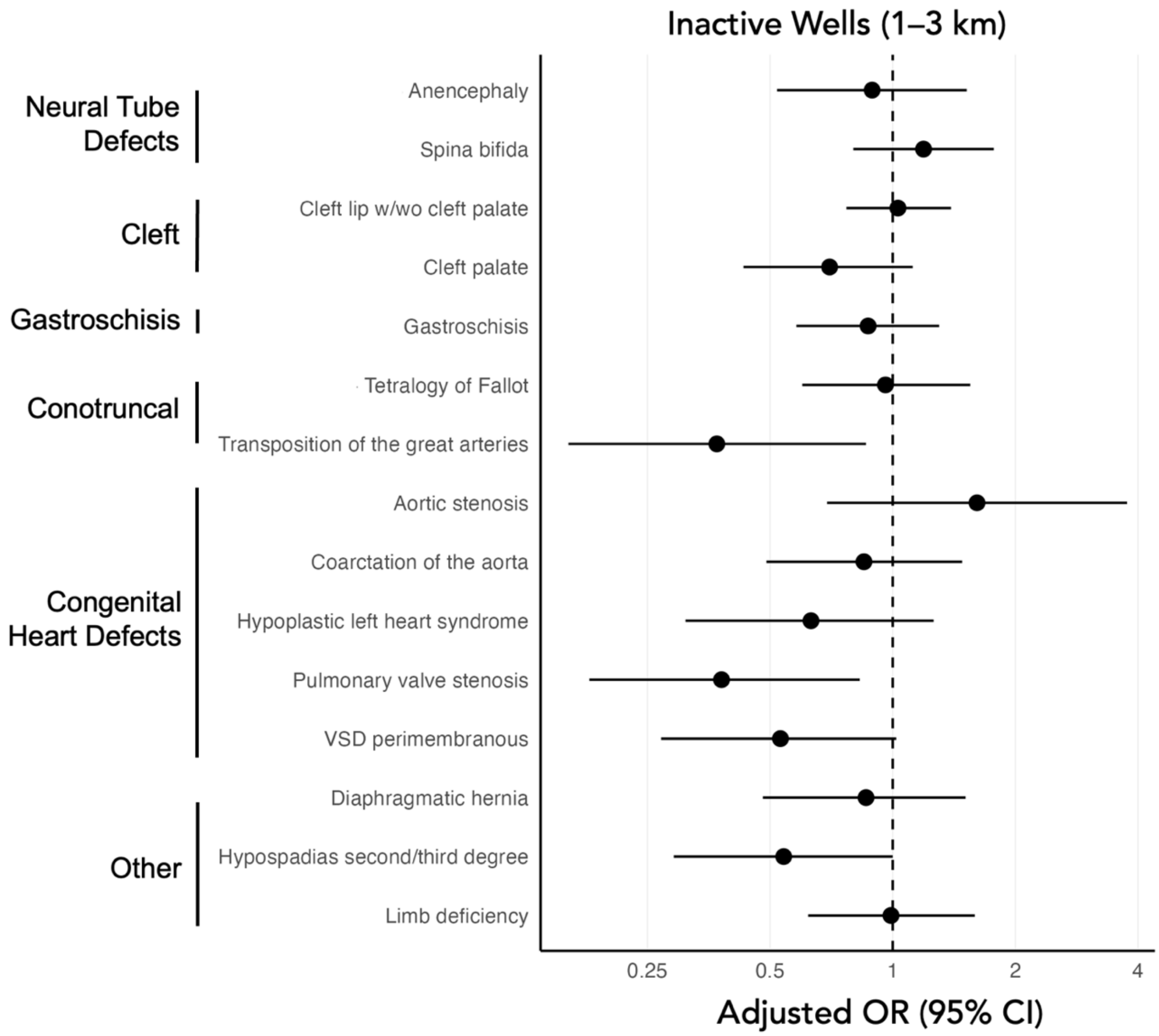

Figure S1. ORs and 95% CIs for exposure to only inactive wells within 1–3 km of the residence. Note that the x-axis is on a log-scale.

Table S1. Adjusted odds ratios (OR) and 95% confidence intervals (CI) for the association between each birth defect phenotype and exposure to active wells within 3 km of the residence and inactive wells only within 1 km of the residence.

				Cases (n)		
**Category**	**Outcome**	**Active Wells (3 km) OR (95% CI)**	**Inactive Wells (1 km) OR (95% CI)**	**Unexposed**	**Exposed Active Wells**	**Exposed Inactive Wells**
Neural Tube Defects	Anencephaly	1.06 (0.57, 1.99)	1.41 (0.52, 3.79)	78	13	5
	Spina bifida	0.93 (0.55, 1.56)	1.71 (0.8, 3.68)	123	19	9
Cleft	Cleft lip w/wo cleft palate	0.91 (0.63, 1.33)	1.75 (1.00, 3.07)	288	43	22
	Cleft palate	0.72 (0.41, 1.28)	2.19 (1.09, 4.4)	126	15	12
Gastroschisis	Gastroschisis	0.5 (0.28, 0.88)	1.09 (0.49, 2.44)	183	16	9
Conotruncal	Tetralogy of Fallot	0.79 (0.42, 1.5)	1.52 (0.62, 3.74)	93	12	6
	Transposition of the great arteries	1.02 (0.52, 1.99)	1.76 (0.66, 4.69)	64	11	5
Congenital Heart Defects	Aortic stenosis	1.58 (0.62, 4.01)	NA	21	6	0
	ASD secundum	0.42 (0.21, 0.85)	1.08 (0.44, 2.65)	128	9	6
	Coarctation of the aorta	0.57 (0.25, 1.26)	1.85 (0.75, 4.56)	75	7	6
	Hypoplastic left heart syndrome	0.51 (0.2, 1.29)	NA	60	5	< 5
	Pulmonary valve stenosis	0.54 (0.21, 1.39)	1.59 (0.58, 4.38)	70	5	5
	VSD perimembranous	0.74 (0.36, 1.51)	1.79 (0.72, 4.44)	77	9	6
Other	Diaphragmatic hernia	1 (0.51, 1.95)	NA	70	11	< 5
	Hypospadias second/third degree	0.59 (0.28, 1.23)	1.38 (0.53, 3.6)	89	9	6
	Limb deficiency	0.96 (0.54, 1.72)	1.7 (0.73, 3.95)	95	15	7

ASD, atrial septal defect; AVSD, atrioventricular septal defect; NOS, not otherwise specified; TAPVR, total anomalous pulmonary venous return; VSD, ventricular septal defect
